# Associations of body mass index with severe outcomes of COVID-19 among critically ill elderly patients: A prospective study

**DOI:** 10.3389/fnut.2023.993292

**Published:** 2023-02-22

**Authors:** Zahra Gholi, Zahra Vahdat Shariatpanahi, Davood Yadegarynia, Hassan Eini-Zinab

**Affiliations:** ^1^Department of Clinical Nutrition and Dietetics, Faculty of Nutrition Sciences and Food Technology, National Nutrition and Food Technology Research Institute, Shahid Beheshti University of Medical Sciences, Tehran, Iran; ^2^Infectious Disease and Tropical Medicine Research Center, Shahid Beheshti University of Medical Science, Tehran, Iran; ^3^Department of Community Nutrition, Faculty of Nutrition and Food Technology, and National Nutrition and Food Technology Research Institute (WHO Collaborating Center), Shahid Beheshti University of Medical Sciences, Tehran, Iran

**Keywords:** COVID-19, obesity, overweight, delirium, critically ill patients, elderly, Body Mass index

## Abstract

**Background and Aim:**

Few studies assessed the associations of overweight and obesity with severe outcomes of coronavirus disease 2019 (COVID-19) among elderly patients. This study was conducted to assess overweight and obesity in relation to risk of mortality, delirium, invasive mechanical ventilation (IMV) requirement during treatment, re-hospitalization, prolonged hospitalization, and ICU admission among elderly patients with COVID-19.

**Methods:**

This was a single-center prospective study that was done on 310 elderly patients with COVID-19 hospitalized in the intensive care unit (ICU). We collected data on demographic characteristics, laboratory parameters, nutritional status, blood pressure, comorbidities, medications, and types of mechanical ventilation at baseline. Patients were followed up during ICU admission and until 45 days after the first visit, and data on delirium incidence, mortality, need for a form of mechanical ventilation, discharge day from ICU and hospital, and re-hospitalization were recorded for each patient.

**Results:**

During the follow-up period, we recorded 190 deaths, 217 cases of delirium, and 35 patients who required IMV during treatment. After controlling for potential confounders, a significant association was found between obesity and delirium such that obese patients with COVID-19 had a 62% higher risk of delirium compared with normal-weight patients (HR: 1.62, 95% CI: 1.02–2.57). This association was not observed for overweight. In terms of other outcomes including ICU/45-day mortality, IMV therapy during treatment, re-hospitalization, prolonged hospitalization, and ICU admission, we found no significant association with overweight and obesity either before or after controlling for potential confounders.

**Conclusion:**

We found that obesity may be a risk factor for delirium among critically ill elderly patients with COVID-19.

## Introduction

Coronavirus disease 2019 (COVID-19) has been a pandemic disease that is caused by severe acute respiratory syndrome coronavirus 2 (SARS-CoV-2) (1). It has become a major threat to global public health during the last 2 years (1). COVID-19 is associated with a high variation in disease severity (2). Young patients usually experience mild-to-moderate symptoms, while elderly patients and those with comorbidities including diabetes (3, 4), cardiovascular diseases (CVDs), cancer, and pulmonary disease are at an increased risk of severe symptoms such as acute respiratory distress syndrome (ARDS) and even death due to COVID-19 pneumonia (2, 5). Therefore, detecting the factors involved in the high severity of disease among the elderly is urgently required.

To date, it has been shown that older age, smoking, taking immunosuppressive drugs such as glucocorticoids and benzodiazepine, and having comorbidities including diabetes, pulmonary diseases, and CVDs are associated with high severity of COVID-19 (6–8). Also, protein–energy malnutrition (PEM) and micronutrient deficiencies such as vitamin D deficiency are associated with a weakening immune system and therefore adversely affect COVID-19 outcomes. Moreover, elevated levels of inflammatory biomarkers may increase the risk of mortality and other severe outcomes in these patients. Recently, great attention has been paid to obesity (9–11). Some studies have shown that obese patients with COVID-19 are at a higher risk of requiring admission to the intensive care unit (ICU) and invasive mechanical ventilation (12, 13). Also, in ICU patients with or without COVID-19, overweight and obesity are associated with an increased risk of ARDS (14). By contrast, several studies indicated that overweight and obesity have a protective effect against mortality among critically ill patients in the ICU (15, 16). Therefore, a potential bidirectional relationship may exist between obesity and COVID-19. Studies have shown an increased SARS-CoV-2 susceptibility in individuals with overweight/obesity, more so in those with coexisting diabetes, as well as an increase in body mass index (BMI) following predominant mild and asymptomatic SARS-CoV-2 infection (17, 18). It has been suggested that a higher metabolic reserve in patients with obesity and differences in pulmonary mechanics and immunological aspects between patients with obesity and normal-weight patients are involved in the protective effect (19). The different effects of obesity on ICU patients are known as the “obesity paradox” (20).

This obesity paradox might be present in critically ill patients with COVID-19, and it is not clear how obesity affects the risk of mortality in these patients. However, the HOPE COVID-19-Registry showed no evidence of the obesity paradox and revealed that increasing BMI was not related to the mortality risk in patients with COVID-19 (21). Some studies have shown a significant association between obesity and mortality due to COVID-19 (22), while others revealed no significant association (23) or even an inverse association (24, 25). In a meta-analysis of 22 studies from seven countries, Zhang et al. (26) reported that obesity is associated with a more severe COVID-19 course but may not be associated with increased mortality. In another meta-analysis, Ho et al. (27) concluded that obesity increased the risk of severe complications, mortality, and infection among patients with COVID-19. In addition, the influence of obesity on other outcomes of critically ill patients with COVID-19 such as delirium and duration of ICU stay has not been studied. Delirium is the most common form of acute brain dysfunction affecting approximately 80% of ICU patients (28). Overall, given the aforementioned points, this study was conducted to assess the associations between obesity and severe outcomes of COVID-19 among critically ill patients.

## Materials and methods

### Study design and participants

This was a single-center prospective study that was conducted in the Khatam hospital, which was a government-designated referral hospital for patients with COVID-19. The location of this hospital was such that patients with COVID-19 from different socioeconomic levels could be admitted to it. This study was conducted from August 2021 to January 2022. We recruited critically ill older (≥65 years) patients with COVID-19 who were hospitalized in the intensive care unit (ICU). SARS-COV-2 infection was diagnosed by reverse transcriptase polymerase chain reaction (RT-PCR) test and also chest CT scan lesions. Based on the classification of the Guidance for Coronavirus Disease 2019 (6th edition), published by the National Health Commission of China (29), we defined critically ill patients with COVID-19 according to the following criteria: (1) respiratory failure requiring a form of mechanical ventilation; (2) septic shock; and (3) having at least one organ failure necessitating monitoring and treatment in the intensive care unit (ICU). Other inclusion criteria were willingness to participate in the study and having an age of ≥65 years. We did not include patients with COVID-19 if (1) they were admitted to the ICU for the second time; (2) they had severe comorbidities including any brain damage and pre-existing end-stage liver disease, end-stage renal disease, and cancer; and (3) they had a history of pre-existing neurodegenerative disorders, mental illness, dementia, and cognitive disorders. Data from these disorders were obtained by evaluating medical records in the hospital. In addition, patients with COVID-19 who died or were discharged within the first 48 h of hospitalization were excluded because of the avoidance of bias in collecting information on complications and reviewing the effectiveness of treatments prescribed in the ICU. In total, 392 elderly patients with COVID-19 were included. We collected data on demographic characteristics, laboratory parameters, nutritional status, blood pressure, comorbidities, medications used for controlling the infection, and types of mechanical ventilation at baseline. Patients were followed up during the ICU admission and also until 45 days after the first visit to the ICU. During the follow-up period, we recorded data on delirium incidence, mortality, need for a form of mechanical ventilation, discharge day from the ICU and hospital, and re-hospitalization for each patient.

### Ethics statement

We took written informed consent from each participant. If a patient was not conscious, the consent was taken from his/her first-degree relatives. Patients were reassured that data collected from medical records would be used for the current study in accordance with privacy laws. The study was approved by the Ethics Committee of Shahid Beheshti University of Medical Sciences, Tehran, Iran (IR.SBMU.NNFTRI.REC.1400.071). We conducted this study based on the ethical standards laid down in the 1964 Declaration of Helsinki and its later amendments.

### Sample size calculation

We calculated the required sample size using Power Analysis Software (PAS). By considering the type 1 error of 5%, study power of 80%, estimated hazard ratio (HR) of 1.2 for mortality, and mortality rate of 60% among critically ill patients with COVID-19, we needed a sample size of 272 elderly patients with COVID-19. However, we recruited 392 patients in the current study to increase study power and consider the probable drop-out.

### Baseline assessment

During the first 24 h of ICU admission, data on demographic characteristics, laboratory parameters, nutritional status, blood pressure, comorbidities, medications used for controlling the infection, and types of mechanical ventilation were collected.

#### Demographic and clinical characteristics

We collected data on age (year), sex (male/female), weight (kg), height (m), marital status (single/married/divorced), having health insurance (yes/no), education (university educated/under-university educated), smoking (non-smokers/ex-smokers/current smokers), systolic and diastolic blood pressure (mmHg), and alcohol consumption (yes/no) by evaluating the hospital’s electronic medical records or questionnaires and also by a direct interview with patients if needed. BMI was determined as weight in kilograms divided by height in meters squared. To collect data on weight, we used data from the medical records of patients. However, these records lacked data on height. Due to the inability of patients to move, the height was estimated by the length of the forearm’s ulna bone. Based on this technique, the patient’s arm was positioned on the shoulder by being bent across the chest to the opposite side. Then, we measured the distance between the conspicuous wrist bone and the elbow bone using a tape measure. The following formula, designed for critically ill patients, was used to estimate height based on gender and ulna bone length: Height (cm) = 153.492 – [7.97 × sex (male = 1, female = 2) + (0.974 × Ulna length (cm)] (30).

In addition, by reviewing the medical records at baseline, we obtained data on comorbidities (yes/no) including pulmonary diseases (i.e., acute pulmonary edema, asthma, bronchitis, chronic obstructive pulmonary disease, pleural effusion, pneumonia, pulmonary mass, pulmonary edema, respiratory tract infection, and sleep apnea syndrome), hyperlipidemia (total cholesterol levels of ≥4.7 mmol/L, triglyceride levels of ≥2.3 mmol/L, or LDL-C levels of ≥4.1 mmol/L), diabetes (2-h plasma glucose ≥200 mg/dL, HbA1c ≥ 6.5%, and fasting plasma glucose ≥126 mg/dL), hypertension (SBP ≥ 140 and DBP ≥ 90), CVDs (i.e., heart failure, left ventricular systolic dysfunction, right heart failure, dysrhythmia, ischemic heart disease, inflammatory heart disease or pericardium, non-ischemic cardiomyopathy, cardiogenic shock, cardiac arrest, and thrombotic disorders), chronic renal failure and liver disease (any type, based on data from medical records), incidence of organ failure from the time of entering in ICU, and ear and eye problems (any type, based on data from medical records). Organ failure was considered the failure of at least one organ to perform typical bodily tasks. This failure comprised at least one of the following: cardiovascular illness, lung failure, acute liver dysfunction, acute renal damage, a wide range of hematological abnormalities, and neurological diseases, as determined by a specialist. During the ICU admission, the incidence of acute kidney injury (AKI), caused by COVID-19 or medications (i.e., Remdesivir), was recorded by reviewing the medical records. AKI was defined as a rise in serum creatinine by 0.3 mg/dL (26.5 μmol/L) or more within 48 h (31). If data from medical records were incomplete for the diagnosis of mentioned diseases, we asked some questions to patients or their relatives to complete the aforementioned information.

The treatment protocols including medications and types of mechanical ventilation [invasive and non-invasive mechanical ventilation (IMV and NIMV), high-flow nasal cannula, and face mask] used for controlling COVID-19 and its symptoms were also recorded. We recorded the drugs that were currently used by patients. By using data on demographic (age), clinical (body temperature, mean arterial pressure, blood pH, heart rate, respiratory rate, oxygen partial pressure, and Glasgow coma scale), and laboratory variables (sodium, potassium, creatinine, hematocrit, and white blood cells), we calculated acute physiology and chronic health examination II (APACHE II) score for each patient. APACHE II scores range between zero and 71, with higher scores indicating a more severe condition. Details on the calculation of APACHE II were published elsewhere (32).

#### Laboratory parameters

On the first day of ICU admission, patients’ medical records were assessed to obtain data on fasting blood sugar (FBS, mg/dL), serum levels of inflammatory biomarkers [C-reactive protein (CRP, mg/L) and interleukin-6 (IL-6, pg./mL)], albumin (g/dL), creatinine (mg/dL), urea (mg/dL), bilirubin (mg/dL), and 25-hydroxy vitamin D3 [25(OH)D3, ng/mL]. Serum levels of electrolytes including magnesium (mEq/L), phosphorous (mg/dL), calcium (mg/dL), sodium (mEq/L), and potassium (mEq/L) were also assessed. We also collected data on hematological factors including white blood cells (neutrophil and lymphocyte, 10^3^/μL), hematocrit (%), and platelet (10^3^/μL).

#### Follow-up

The incidence of delirium and the need for a form of mechanical ventilation (yes/no), particularly invasive ventilation, were recorded during the ICU admission. Delirium was diagnosed based on the Confusion Assessment Method for the Intensive Care Unit (CAM-ICU) (33). Accordingly, delirium has four features: (1) acute onset of changes or fluctuations in the course of mental status, (2) inattention, (3) disorganized thinking, and (4) an altered level of consciousness (other than alert). Patients were delirious if they had features 1 and 2 plus either feature 3 or 4. In the present study, delirium was evaluated every day using the CAM-ICU by an experienced ICU physician. To facilitate the assessment of acute onset or fluctuation of mental status changes, patients were followed up daily with the Glasgow coma scale. In addition to delirium, we recorded the occurrence of mortality (yes/no) during the ICU admission. After the ICU discharge, patients were admitted to the other wards of the hospital. Therefore, we followed patients in the hospital until they were discharged. Furthermore, the length of ICU and hospital stays was recorded for each patient. After the hospital discharge, we had phone contact with patients or their relatives every week, until 45 days after the baseline, to record probable death and re-hospitalization. ICU admission ≥7 days was considered a prolonged stay in ICU and hospitalization ≥14 days was a prolonged stay in the hospital.

#### Statistical analysis

We first categorized elderly patients with COVID-19 based on BMI [normal-weight (BMI < 25), overweight (25 ≤ BMI < 30), and obesity (BMI ≥ 30)], according to the recommended classification by the World Health Organization (34, 35). Then, we compared continuous variables across categories of BMI using one-way ANOVA if the distribution of those variables was normal. For the non-normally distributed continuous variables, we used the Kruskal–Wallis test for comparison. To assess the distribution of categorical variables across categories of BMI, we used the Chi-square test. In order to analyze the associations of BMI categories with mortality, delirium, and IMV therapy during treatment, we used univariable and multivariable Cox proportional hazards models. In the time-to-event analysis, follow-up time was considered as the day that outcome occurred or the day that the patient was followed up. To assess the associations of BMI categories with prolonged stay in ICU (≥7 days) or hospital (≥14 days) and odds of re-hospitalization after discharge, we used univariable and multivariable binary logistic regression. In the adjusted models, we controlled for age, gender, taking benzodiazepine during ICU admission, and vitamin D and IL-6 levels. To identify potential confounders, we calculated the magnitude of confounding for each variable as the percent difference between the crude and adjusted measures of association (Supplementary Tables S1, S2). The following formula was used for the relative risk estimates:

Magnitude of confounding (%) = $\frac{RR_{crude-RR_{adjusted}}}{RR_{adjusted}}$×100.

If the value was ≥10% for a variable, that variable was considered a confounding variable. By this approach, we found that age, benzodiazepine intake, and IL-6 levels (only adjusted for death during ICU admission) were confounders for the associations of BMI with delirium, IMV therapy, and COVID-19 mortality. Also, for the associations of BMI with re-hospitalization and prolonged hospital/ICU stays, we considered age, gender, benzodiazepine intake, and vitamin D levels as confounders. In all analyses, normal-weight patients with COVID-19 were considered as a reference group. All statistical analyses were done using the SPSS software version 18 (SPSS, Inc. Chicago, IL, USA). *p* < 0.05 was considered significant.

## Results

**Figure 1:**
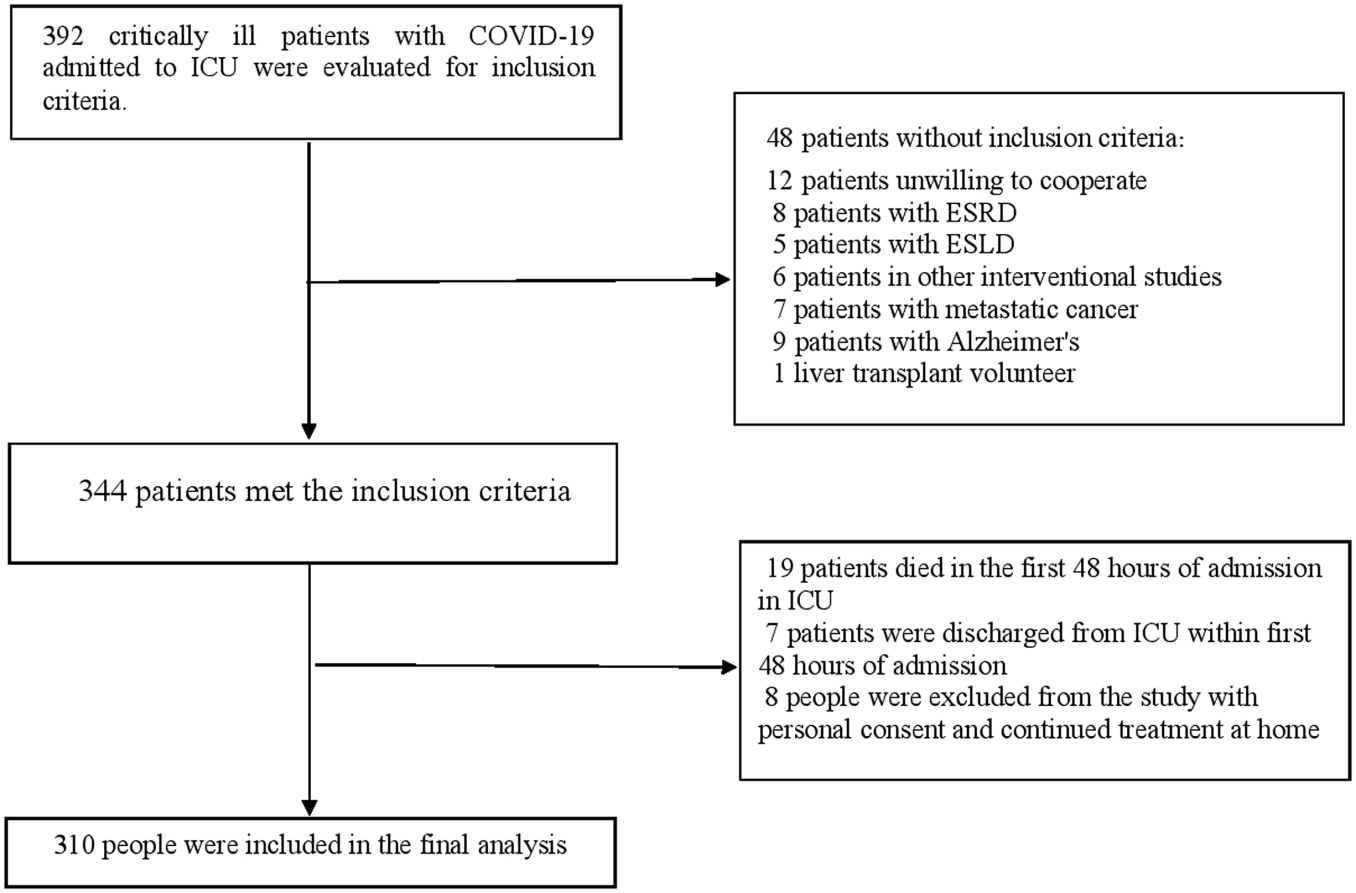
Study flow diagram.

Of the 392 critically ill elderly patients with Covid-19 admitted to the intensive care unit, 48 patients did not meet the inclusion criteria as shown in Figure 1. Out of 344 patients who met the inclusion criteria, 19 patients died within the first 48 hours of admission to the ICU and 7 patients were discharged from the intensive care unit within the first 48 hours of admission. During the follow-up period of the patients in the ICU, 8 patients were discharged from the ICU with personal consent to continue the treatment process at home. Therefore, the data of 310 patients were included in the final analysis. All patients had received antiviral and antibiotic drugs. Antiviral drugs included remdesivir, favipiravir, tocilizumab, and lopinavir/ritonavir, which were available in Iranian hospitals. During the 45-day follow-up, 190 (61.3%) COVID-19 deaths were recorded among the baseline 310 patients. In addition, during the ICU admission, 217 (70.0%) cases of delirium and 53 (17.1%) patients who required IMV therapy from the beginning of treatment were found in the ICU. Also, during the 45-day follow-up, 65 (21.0%) patients were hospitalized for the second time.

The baseline characteristics of patients across categories of BMI are shown in Tables 1, 2. Patients with obesity had lower ages and were more likely to consume vasopressors compared to those with normal weight. In terms of other variables including government health insurance, laboratory parameters, comorbidities, drug use, duration of hospital and ICU stays, and types of ventilation, we found no significant difference.

**Table 1:** Baseline characteristics of critically ill elderly patients with COVID-19 across categories of BMI.

	Total	Normal	Overweight	Obesity	*p*-value*
*n*	310	79	191	40	
Demographic characteristics					
Age, y	73.29 ± 6.91	76.90 ± 6.87	72.19 ± 6.36	71.37 ± 7.11	<0.001
Weight, kg	72.77 ± 10.50	61.80 ± 6.43	74.39 ± 7.01	87.00 ± 9.24	<0.001
BMI, kg/m^2^	26.88 ± 3.28	23.17 ± 1.29	27.19 ± 1.38	32.79 ± 2.92	<0.001
Female, %	128 (41.3)	32 (40.5)	73 (38.2)	23 (57.5)	0.08
Smokers, %	82 (26.5)	21 (26.6)	49 (25.7)	12 (30)	0.85
Government health insurance,%	252 (81.3)	63 (79.7)	155 (81.2)	34 (85)	0.78
Alcohol intake, %	24 (7.7)	6 (7.6)	16 (8.4)	2 (5)	0.76
Comorbidities					
Pulmonary disease, %	77 (24.8)	23 (29.1)	46 (24.1)	8 (20)	0.51
Hyperlipidemia, %	124 (40.0)	38 (48.1)	69 (36.1)	17 (42.5)	0.17
Diabetes, %	141 (45.5)	37 (46.8)	90 (47.1)	14 (35)	0.36
Hypertension, %	155 (50.0)	50 (63.3)	100 (52.4)	20 (50)	0.20
CVD, %	139 (44.8)	43 (54.4)	79 (41.4)	17 (42.5)	0.13
Chronic renal disease, %	111 (35.8)	31 (39.2)	68 (35.6)	12 (30.0)	0.60
Liver disease, %	28 (9.0)	9 (11.4)	15 (7.9)	4 (10.0)	0.63
Stroke, %	17 (5.5)	7 (8.9)	9 (4.8)	1 (2.6)	0.28
Organ failure, %^a^	149 (48.1)	36 (45.6)	92 (48.2)	21 (52.5)	0.77
Ear problems, %	27 (8.7)	9 (11.4)	17 (8.9)	1 (2.5)	0.26
Eye problems, %	17 (5.5)	6 (7.6)	10 (5.2)	1 (2.5)	0.49
Medication					
Propofol, %	20 (6.5)	3 (3.8)	16 (8.4)	1 (2.5)	0.20
Opioid drugs, %	182 (58.7)	51 (64.6)	109 (57.1)	22 (55.0)	0.46
Glucocorticoids, %	203 (65.5)	55 (69.6)	120 (62.8)	28 (70.0)	0.45
Benzodiazepine, %	219 (70.6)	59 (74.7)	137 (71.7)	23 (57.5)	0.13
Vasopressor, %	145 (46.8)	34 (43.0)	83 (43.5)	28 (70)	0.007
Oxygen therapy at baseline					0 ()
IMV, %	20 (6.5)	3 (3.8)	16 (8.4)	1 (2.5)	0.20
NIV, %	185 (59.7)	48 (60.8)	113 (59.2)	24 (60.0)	0.97
High flow nasal cannula, %	7 (2.3)	5 (6.3)	1 (0.5)	1 (2.5)	0.01
Face mask, %	183 (59.0)	41 (51.9)	115 (60.2)	27 (67.5)	0.22

Data are presented as mean ± SD for normally distributed continuous variables, median (interquartile range) for non-normally distributed continuous variables, and percent for categorical variables.

BMI: body mass index, CVD: cardiovascular disease, IMV: invasive mechanical ventilation, NIV: non-invasive ventilation, SD: standard deviation.

a Considered as the incidence of failure of ≥ 2 organs.

* Obtained from the one-way ANOVA (normally distributed continuous variables), the Kruskal–Wallis test (non-normally distributed continuous variables), or the chi-square test (categorical variables).

**Table 2:** Clinical characteristics of critically ill elderly patients with COVID-19 across categories of BMI.

	Total	Normal	Overweight	Obesity	*p*-value*
*n*	310	79	191	40	
Hematology					
WBC, 10^3^/μL	9.37 ± 4.51	9.14 ± 4.94	9.39 ± 4.26	9.75 ± 4.84	0.78
Neutrophil, 10^3^/μL	83.43 ± 8.80	82.59 ± 9.34	83.99 ± 8.30	82.42 ± 10.01	0.38
Lymphocyte, 10^3^/μL	12.20 ± 12.17	13.15 ± 12.76	12.15 ± 12.64	10.72 ± 8.29	0.59
Neutrophil/lymphocyte ratio	12.04 ± 10.24	12.80 ± 14.58	11.59 ± 7.99	12.65 ± 9.56	0.48
Albumin, g/dL	3.05 ± 0.65	2.92 ± 0.67	3.08 ± 0.66	3.14 ± 0.57	0.11
Biochemical assessment					
CRP, mg/L	87.41 ± 47.31	85.64 ± 44.29	88.66 ± 48.13	84.95 ± 50.02	0.83
IL6, pg./mL	159.47 ± 216.41	142.91 ± 137.59	164.80 ± 241.15	166.69 ± 221.72	0.73
Creatinine, mg/dL	1.40 ± 0.62	1.40 ± 0.63	1.36 ± 0.50	1.56 ± 1.02	0.20
FBS, mg/dL	168.89 ± 53.83	170.87 ± 55.78	171.17 ± 53.02	154.20 ± 52.85	0.18
Vitamin D, ng/mL	30.03 ± 8.76	29.55 ± 8.19	30.32 ± 9.06	29.58 ± 8.56	0.75
Bilirubin, mg/dL	0.83 ± 1.14	0.80 ± 0.91	0.89 ± 1.32	0.63 ± 0.32	0.38
Urea, mg/dL	27.69 ± 16.15	27.14 ± 16.33	27.08 ± 13.71	31.63 ± 24.46	0.25
Magnesium, mEq/L	1.98 ± 0.39	1.97 ± 0.37	1.98 ± 0.40	2.01 ± 0.36	0.90
Calcium, mg/dL	8.11 ± 0.57	8.13 ± 0.49	8.13 ± 0.61	8.01 ± 0.55	0.50
Sodium, mEq/L	136.43 ± 8.85	135.46 ± 16.06	136.59 ± 4.21	137.55 ± 3.80	0.44
Potassium, mmol/L	4.00 ± 0.69	4.08 ± 0.84	3.95 ± 0.65	4.07 ± 0.58	0.36
Blood pressure					
SBP, mmHg	139.09 ± 22.20	139.44 ± 23.49	139.99 ± 22.00	134.10 ± 20.29	0.30
DBP, mmHg	81.28 ± 15.30	80.50 ± 15.01	82.17 ± 15.18	78.59 ± 16.41	0.35
Mean arterial pressure, mmHg	100.55 ± 16.69	100.14 ± 16.92	101.44 ± 16.53	97.09 ± 16.94	0.31
Outcomes during follow-up					
IMV therapy,%	53 (17.1)	20 (25.3)	30 (15.7)	3 (7.5)	0.03
Delirium,%	217 (70.0)	54 (68.4)	133 (69.6)	30 (75.0)	0.74
Death during ICU admission,%	132 (42.6)	42 (53.2)	76 (39.8)	14 (35)	0.07
Death during 45 days,%	190 (61.3)	52 (65.8)	117 (61.3)	21 (52.5)	0.23
Re-hospitalization,%	65 (21.0)	19 (24.1)	38 (19.9)	8 (20)	0.73
Acute renal failure, %	82 (26.5)	17 (21.5)	53 (27.7)	12 (30)	0.49
Hospitalization					
Length of hospital stay (day)	14 (10–19)	13 (10–18)	14 (10–19)	16 (12–21)	0.13
Length of ICU stay (day)	8 (6–10)	8 (6–10)	8 (6–10)	8 (5–10)	0.96
APACHE II score	17 (11–21)	17 (11–21)	17 (12–21)	16 (11–19)	0.54

Data are presented as mean ± SD for normally distributed continuous variables, median (interquartile range) for non-normally distributed continuous variables, and percent for categorical variables.

BMI: body mass index, WBC: white blood cell, IL-6: interleukin-6, CRP: C-reactive protein, FBS: fasting blood sugar, SBP: systolic blood pressure, DBP: diastolic blood pressure, CVD: cardiovascular disease, IMV: invasive mechanical ventilation, SD: standard deviation, APACHE II: acute physiology and chronic health examination II.

* Obtained from the one-way ANOVA (normal-distributed continuous variables), the Kruskal–Wallis test (non-normally distributed continuous variables), or the chi-square test (categorical variables).

Multivariable-adjusted HRs and 95% confidence intervals (CIs) of delirium, COVID-19 mortality, and IMV therapy during treatment across categories of baseline BMI among critically ill elderly patients with COVID-19 are indicated in Table 3. We found no significant association between overweight/obesity and delirium among elderly patients with COVID-19 (overweight; HR: 1.06, 95% CI: 0.77–1.46, obesity; HR: 1.27, 95% CI: 0.81–1.99). However, after controlling for potential confounders including age and benzodiazepine intake, a significant association was seen for obesity; such that obese patients with COVID-19 had a 62% higher risk of delirium compared with normal-weight patients (HR: 1.62, 95% CI: 1.02–2.57). Such an association was not seen for overweight. Before and after taking potential confounders into account, no significant association was found between BMI categories (overweight and obesity) and risk of IMV requirement during treatment. Regarding the risk of mortality during ICU admission and 45 days after the baseline, we found no significant association with overweight or obesity either before or after controlling for potential confounders.

**Table 3:** Hazard ratios for some outcomes of critically ill elderly patients with COVID-19 across categories of BMI.

	Normal	Overweight	Obesity
Delirium			
Cases	54	133	30
Unadjusted	1.00	1.06 (0.77–1.46)	1.27 (0.81–1.99)
Adjusted model^b^	1.00	1.20 (0.86–1.67)	1.62 (1.02–2.57)
IMV therapy during treatment^a^			
Cases	20	30	3
Unadjusted	1.00	0.66 (0.37–1.17)	0.30 (0.90–1.02)
Adjusted model^b^	1.00	0.95 (0.52–1.74)	0.56 (0.16–1.92)
Death during 45 days			
Cases	52	117	21
Unadjusted	1.00	0.90 (0.65–1.25)	0.73 (0.44–1.21)
Adjusted model^b^	1.00	1.16 (0.82–1.64)	1.10 (0.65–1.86)
Death during ICU admission			
Cases	42	76	14
Unadjusted	1.00	0.79 (0.54–1.16)	0.67 (0.37–1.23)
Adjusted model^b^	1.00	0.97 (0.65–1.45)	0.96 (0.52–1.79)

Data are presented as HR (95% CI).

BMI: body mass index, IMV: invasive mechanical ventilation, HR: hazard ratio, ICU: intensive care unit.

a With considering IMV therapy at baseline.

b Adjusted for age, benzodiazepine intake, and IL6 levels (only adjusted for death during ICU admission).

HRs were obtained from the Cox regression analysis.

Multivariable-adjusted odds ratios (ORs) and 95% CIs for re-hospitalization and prolonged stay in ICU and hospital across categories of BMI are presented in Table 4. Overweight and obesity were not significantly associated with re-hospitalization and prolonged stay in ICU and hospital. These associations remained non-significant after taking potential confounders into account [re-hospitalization (overweight; OR: 0.98, 95% CI: 0.48–1.99, obesity; OR: 1.12, 95% CI: 0.41–3.06), prolonged stay in ICU (overweight; OR: 1.01, 95% CI: 0.56–1.81, obesity; OR: 1.12, 95% CI: 0.49–2.56), and prolonged stay in ICU and hospital (overweight; OR: 0.99, 95% CI: 0.55–1.78, obesity; OR: 1.57, 95% CI: 0.67–3.64)].

**Table 4:** Odds ratios for re-hospitalization and prolonged stay in ICU and hospital across categories of BMI in critically ill elderly patients with COVID-19.

	Normal	Overweight	Obesity
Hospital stay ≥ 14 days			
Cases	38	97	26
Unadjusted	1.00	1.11 (0.65–1.88)	2.00 (0.91–4.39)
Adjusted model^a^	1.00	0.99 (0.55–1.78)	1.57 (0.67–3.64)
ICU stay≥7 days			
Cases	50	118	25
Unadjusted	1.00	0.93 (0.54–1.61)	0.96 (0.44–2.12)
Adjusted model^b^	1.00	1.01 (0.56–1.81)	1.12 (0.49–2.56)
Re-hospitalization			
Cases	19	38	8
Unadjusted	1.00	0.78 (0.41–1.46)	0.79 (0.31–2.00)
Adjusted model^c^	1.00	0.98 (0.48–1.99)	1.12 (0.41–3.06)

Data are presented as OR (95% CI).

BMI: body mass index, ICU: intensive care unit, OR: odds ratio

a Adjusted for age, gender, and benzodiazepine intake.

b Adjusted for gender.

c Adjusted for vitamin D levels.

ORs were obtained from the binary logistic regression.

## Discussion

Since obesity and overweight are similar in nature, we discussed both in the same manner, particularly when the findings of both conditions were similar. In the current study, we found that elderly patients with COVID-19 with obesity had an increased risk of delirium than those with normal weight. This association was not seen for overweight. In terms of other outcomes including ICU and 45-day mortality, IMV therapy during treatment, prolonged stay in ICU and hospital, and odds of re-hospitalization, we observed no significant association with overweight and obesity.

Our study represented the overall mortality rate of 42.3% among elderly patients with COVID-19 admitted to the ICU. Compared with the rate obtained from previous studies (36, 37), it seems to be high. In a meta-analysis, Qian et al. (38) indicated a prevalence of 32% for COVID-19 mortality among critically ill patients. The higher prevalence of mortality in the current study might be due to the age range of participants who were 65 years and older. It has been shown that older age is the main risk factor for COVID-19 mortality (39–41). It should be noted that the prevalence of 32% in the study of Qian et al. was obtained by assessing different age groups.

Delirium is an acute disturbance of consciousness that is associated with mental disorders such as sleep disorders, changes in cognitive functions, anxiety, fear, and irritability (42, 43). Delirious patients in ICU have an increased risk of mortality, longer ICU hospitalizations, extended periods of mechanical ventilation, and long-term cognitive and functional deficits. Known risk factors for developing delirium include aging, baseline cognitive impairment, comorbidities (particularly respiratory disease), frailty, sepsis, prolonged mechanical ventilation, and major surgery (44–46). However, few studies investigated the link between obesity and delirium in critically ill patients. In the current study, we found that elderly obese patients with COVID-19 had a higher risk of delirium compared with normal-weight patients. Such an association was not seen for overweight. In a study on 9,189 adults, Anand et al. (47) reported that obesity was associated with a reduced cognitive score indicating lower cognitive function. In contrast, Lachmann et al. (48) reported that diabetes, but not obesity or hypertension, was associated with an increased risk of postoperative cognitive dysfunction in older people. In a retrospective cohort study, a high BMI was independently associated with a lower frequency of acute delirium in ICU patients with septic shock (49). Discrepant findings might be explained by the different ages and the different medications of study participants in previous studies. For instance, the administration of analgesic medications, some anticholinergic drugs, and benzodiazepine infusions for mechanical ventilation is associated with a higher risk of delirium. It should be noted that medications among the patients who participated in the current study were not different across categories of BMI. In addition, different cognitive and physical reserves of participants are other reasons for the observed discrepancy among previous studies on the link between obesity and cognitive dysfunction.

The mechanism involved in the association between obesity and delirium in ICU patients with COVID-19 infection is unclear. Recent studies have shown that obese patients with COVID-19 have severe symptoms and more need for IMV compared with normal-weight patients (12, 50). In addition, obesity causes mechanical disturbances because abdominal thrusts increase inter-abdominal pressure, which makes it difficult for the lungs to breathe (51). In addition, hospitalized patients should be lying down, particularly in a supine position. This position in patients with obesity makes breathing more difficult (51). Therefore, patients with obesity commonly develop hypoventilation and sleep apnea syndromes with hypoxic and hypercapnic ventilatory responsiveness (52). This condition disrupts the levels of oxygen and CO2 in the blood, induces cerebral oxygen desaturation, and can cause delirium in patients with obesity (53). It should be noted that in the current study, patients with obesity (7.5%) used IMV less frequently than normal-weight patients (25.3%) and this may increase the rate of cerebral oxygen desaturation and might be a reason for the increased odds of delirium among patients with obesity. Another proposed mechanism is the effect of obesity on cognitive function. In a review article, Miller et al. (54) concluded that obesity-induced inflammation (particularly elevated circulating IL-12 and IL-6) was associated with disruption to cognitive function mediated by brain regions such as the hippocampus, amygdala, and reward-processing centers.

In the current study, we found no significant association between BMI categories and IMV requirement among elderly patients with COVID-19 hospitalized in ICU. In line with our findings, Rovirosa et al. (55) showed that patients with obesity and overweight, according to the WHO classification, had no significant association with requiring intubation and IMV in patients with COVID-19. In a study in the US, Kompaniyets et al. (50) reported that overweight and obesity were risk factors for IMV in patients with COVID-19. The study by Kim et al. (56) showed that overweight and all classes of obesity were associated with increased odds of IMV. That study showed that the use of IMV in patients who are overweight and with obesity may be affected by clinical bias toward early intervention based on proven pulmonary complications in patients with obesity. This finding is limited in generalizability due to the differences between the patient populations. Limitations on clinical information include the severity of dyspnea, resuscitation and/or intubation status, or the reason for clinical decision-making to explain which patients were intubated. The results of the CORONADO study, with a large population and good phenotypes of COVID-19 individuals with diabetes admitted to the hospital ward and ICU, showed that the relationship between IMV and BMI appeared with overweight (57). In a case–control study, Ferreira et al. (58) reported that the need for IMV was higher among patients with COVID-19 if they were obese. Different medications, different cutoff points used for the definition of obesity, and different quality of previous studies are probable reasons for the observed discrepancy. In addition, limited facilities in hospitals might be another reason. On the other hand, a limited number of hospital beds with ventilators and not using them for qualified patients may affect the risk estimates obtained from the current and previous studies. However, it must be kept in mind that the hospital where we recruited patients with COVID-19 for the current study had 98 ICU beds and all of them had ventilators for IMV therapy. Therefore, there was no limitation for IMV therapy for patients admitted to ICU. However, because of the low number of ICU beds in that hospital, IMV therapy may not be done for some qualified patients admitted to other wards of the hospital.

Regarding COVID-19 mortality and prolonged hospital stay, no significant association was seen between overweight and obesity in the current study. In agreement with our findings, Pouwels et al. (59) reported that obesity was not related to 28-day mortality and duration of ICU and hospital stay among critically ill patients with COVID-19 infection. In contrast, Kompaniyets et al. (50) indicated that higher BMI in patients with COVID-19 was associated with an increased risk of mortality, hospitalization, and ICU admission. Another study revealed that obesity was an independent risk and prognostic factor for the disease severity and the requirement for advanced medical care in patients with COVID-19 (60). The discrepant findings on obesity and COVID-19 mortality might be due to the obesity paradox. Al-Salameh et al. (61) study showed that the relative risk of transfer to ICU and occurrence of some outcomes, including intubation for mechanical ventilation, ARDS, and acute renal injury, were high in the overweight group, but without the risk of mortality, which indicates the “survival paradox of obesity”. Previous studies on ICU patients have shown a J-shaped association between BMI and mortality, with overweight and moderate obesity being protective compared with a normal BMI or more severe obesity (62). This is in line with our findings, in which a non-significant inverse association was seen between overweight and ICU mortality among patients with COVID-19. Despite this protective effect regarding mortality, it has been shown that obesity among ICU patients increases the risk of infection and respiratory and cardiovascular complications (62). These complications are associated with an increased risk of mortality among ICU patients (63). In addition, using different cutoff points for the definition of overweight and obesity might be involved in the discrepant findings. In terms of different findings on the link between overweight/obesity and prolonged hospitalization, we may justify different treatment protocols and different hospital admission capacities in different countries. Because of a high incidence of COVID-19 infection and limited hospital beds, patients may be discharged prematurely. Therefore, the lack of significant association between overweight/obesity and prolonged hospitalization should be considered with caution. Further studies are needed to substantiate these findings.

This study had some strengths. To the best of our knowledge, this was the first study that examined the link between obesity and the risk of delirium among critically ill elderly patients with COVID-19. The prospective design of our study and controlling for potential confounders were other strengths. Our present study was subjected to some limitations. First, the sample size of this study did not allow us to perform subgroup analyses based on gender and other important variables. In addition, because of the low sample size, we had a lower number of patients in the normal-weight and obese groups compared with the overweight group. Second, the number of patients in the obese group was too small which may reduce the robustness of the analysis result. Third, although we extracted data on height and weight values from medical chart records, some values might have been self-reported, which may lead to measurement bias. Fourth, even though potential confounders had been adjusted in the analysis, our results might be still affected by residual confounders such as lifestyle information and therapeutic protocols used for controlling COVID-19 infection. In addition, the low number of nurses and physicians in the hospital may affect the quality of health services and consequently the risk estimates obtained in the current study. Fifth, we excluded patients with cancer, end-stage liver disease, and end-stage kidney disease. These patients usually have obesity and severe outcomes of COVID-19. Therefore, this exclusion may attenuate the risk estimates calculated for the association between obesity and clinical outcomes of COVID-19. This may also explain the non-significant association between obesity and COVID-19 mortality in the current study.

In conclusion, we found that elderly patients with COVID-19 with obesity have an increased risk of delirium compared with normal-weight patients. However, overweight was not significantly associated with the risk of delirium. Also, overweight and obesity were not significantly associated with other outcomes of elderly patients with COVID-19 such as IMV requirement, ICU/45-day mortality, and prolonged hospitalization. Further studies with higher sample sizes and considering a wide range of confounders are needed to confirm our findings.

### What is already known on this subject?

Previous studies presented inconsistent results on the association between obesity and COVID-19 mortality. Few studies have been done on elderly patients. Also, the influence of obesity on other outcomes of critically ill patients with COVID-19 such as delirium and duration of ICU stay has not been studied.

### What this study adds?

We found that obese elderly patients with COVID-19 have an increased risk of delirium compared with normal-weight patients. Regarding other outcomes including IMV requirement, death, prolonged hospitalization, and ICU admission, we found no significant association with overweight or obesity among elderly patients with COVID-19.

## Data availability statement

The raw data supporting the conclusions of this article will be made available by the authors, without undue reservation.

## Ethics statement

The studies involving human participants were reviewed and approved by Ethics Committee of Shahid Beheshti University of Medical Sciences, Tehran, Iran. The patients/participants provided their written informed consent to participate in this study. Written informed consent was obtained from the individual(s) for the publication of any potentially identifiable images or data included in this article.

## Author contributions

ZGh, DY, HEZ, and ZVSh designed the research project. ZGh and ZVSh conducted the research; ZGh analyzed data; ZGh and ZVSh wrote the paper; ZGh and ZVSh had primary responsibility for final content. All authors read and approved the final manuscript.

## Funding

This study was funded by the Shahid Beheshti University of Medical Sciences, Tehran, Iran. The funder had no role in the design and conduct of the study; collection, management, analysis, and interpretation of the data; preparation, review, or approval of the manuscript; or the decision to submit the manuscript for publication.

## Conflict of interest

The authors declare that the research was conducted in the absence of any commercial or financial relationships that could be construed as a potential conflict of interest.

## Publisher’s note

All claims expressed in this article are solely those of the authors and do not necessarily represent those of their affiliated organizations, or those of the publisher, the editors and the reviewers. Any product that may be evaluated in this article, or claim that may be made by its manufacturer, is not guaranteed or endorsed by the publisher.
