# Supplementary material for: Associations of body mass index with severe outcomes of COVID-19 among critically ill elderly patients: A prospective study
Source: Front Nutr. 2023 Feb 22;10:993292. doi: 10.3389/fnut.2023.993292 (PMC9994813; doi:10.3389/fnut.2023.993292)
Supplement: Supplementary file 1 [file Table_1.docx]

**Supplementary Table 1**: Confounding assessment on the associations of body mass index with some outcomes of critically ill elderly COVID-19 patients

|  |  | Overweight | |  | Obesity | | Results |
| --- | --- | --- | --- | --- | --- | --- | --- |
|  |  | RR (95 CI) | ΔRR (%) |  | RR (95 CI) | ΔRR (%) |  |
| Delirium | |  |  |  |  |  |  |
|  | Crude model | 1.06 (0.82-1.99) | - |  | 1.27 (0.82-1.99) | - |  |
|  | Model 1 with age | 1.17 (0.89-2.25) | 9 |  | 1.42 (0.89-2.25) | 10 | Confounding |
|  | Model 2 with gender | 1.07 (0.81-1.97) | 1 |  | 1.26 (0.81-1.97) | 1 | Not-confounding |
|  | Model 3 with smoking | 1.07 (0.83-2.04) | 1 |  | 1.31 (0.83-2.04) | 2 | Not-confounding |
|  | Model 4 with history of pulmonary diseases | 1.07 (0.84-2.05) | 1 |  | 1.31 (0.84-2.05) | 3 | Not-confounding |
|  | Model 4 with glucocorticoids | 1.07 (0.81-1.99) | 1 |  | 1.27 (0.81-1.99) | 0 | Not-confounding |
|  | Model 5 with benzodiazepine | 1.11 (0.95-2.34) | 4 |  | 1.49 (0.95-2.34) | 15 | Confounding |
|  | Model 6 with neutrophil levels | 1.08 (0.82-2.00) | 2 |  | 1.28 (0.82-2.00) | 0 | Not-confounding |
|  | Model 7 with albumin levels | 1.08 (0.83-2.04) | 1 |  | 1.30 (0.83-2.04) | 2 | Not-confounding |
|  | Model 8 with vitamin D levels | 1.09 (0.83-2.06) | 3 |  | 1.31 (0.83-2.06) | 3 | Not-confounding |
|  | Model 8 with IL-6 levels | 1.05 (0.80-1.96) | 1 |  | 1.26 (0.80-1.96) | 1 | Not-confounding |
| IMV therapy during treatment | |  |  |  |  |  |  |
|  | Crude model | 0.66 (0.09-1.02) | - |  | 0.30 (0.09-1.02) | - |  |
|  | Model 1 with age | 0.87 (0.13-1.48) | 24 |  | 0.43 (0.13-1.48) | 30 | Confounding |
|  | Model 2 with gender | 0.65 (0.10-1.12) | 2 |  | 0.33 (0.10-1.12) | 8 | Not-confounding |
|  | Model 3 with smoking | 0.68 (0.09-1.03) | 2 |  | 0.30 (0.09-1.03) | 0 | Not-confounding |
|  | Model 4 with history of pulmonary diseases | 0.70 (0.09-1.08) | 6 |  | 0.32 (0.09-1.08) | 5 | Not-confounding |
|  | Model 4 with glucocorticoids | 0.72 (0.10-1.09) | 7 |  | 0.32 (0.10-1.09) | 6 | Not-confounding |
|  | Model 5 with benzodiazepine | 0.72 (0.12-1.38) | 8 |  | 0.41 (0.12-1.38) | 25 | Confounding |
|  | Model 6 with neutrophil levels | 0.66 (0.09-1.01) | 1 |  | 0.30 (0.09-1.01) | 1 | Not-confounding |
|  | Model 7 with albumin levels | 0.67 (0.09-1.05) | 1 |  | 0.31 (0.09-1.05) | 3 | Not-confounding |
|  | Model 8 with vitamin D levels | 0.64 (0.08-0.95) | 3 |  | 0.28 (0.08-0.95) | 8 | Not-confounding |
|  | Model 8 with IL-6 levels | 0.67 (0.09-1.04) | 1 |  | 0.31 (0.09-1.04) | 1 | Not-confounding |
| Death during 45 days | |  |  |  |  |  |  |
|  | Crude model | 0.90 (0.44-1.21) | - |  | 0.73 (0.44-1.21) | - |  |
|  | Model 1 with age | 1.14 (0.55-1.57) | 21 |  | 0.93 (0.55-1.57) | 21 | Confounding |
|  | Model 2 with gender | 0.90 (0.44-1.22) | 0 |  | 0.73 (0.44-1.22) | 0 | Not-confounding |
|  | Model 3 with smoking | 0.91 (0.44-1.21) | 2 |  | 0.73 (0.44-1.21) | 0 | Not-confounding |
|  | Model 4 with history of pulmonary diseases | 0.93 (0.47-1.29) | 3 |  | 0.78 (0.47-1.29) | 6 | Not-confounding |
|  | Model 4 with glucocorticoids | 0.91 (0.44-1.21) | 2 |  | 0.73 (0.44-1.21) | 0 | Not-confounding |
|  | Model 5 with benzodiazepine | 0.93 (0.53-1.48) | 4 |  | 0.89 (0.53-1.48) | 18 | Confounding |
|  | Model 6 with neutrophil levels | 0.91 (0.46-1.27) | 2 |  | 0.76 (0.46-1.27) | 4 | Not-confounding |
|  | Model 7 with albumin levels | 0.96 (0.48-1.34) | 6 |  | 0.80 (0.48-1.34) | 9 | Not-confounding |
|  | Model 8 with vitamin D levels | 0.84 (0.40-1.12) | 6 |  | 0.67 (0.40-1.12) | 9 | Not-confounding |
|  | Model 8 with IL-6 levels | 0.88 (0.42-1.13) | 3 |  | 0.67 (0.40-1.13) | 8 | Not-confounding |
| Death during ICU admission | |  |  |  |  |  |  |
|  | Crude model | 0.79 (0.37-1.23) | - |  | 0.67 (0.37-1.23) | - |  |
|  | Model 1 with age | 0.90 (0.42-1.45) | 12 |  | 0.78 (0.42-1.45) | 14 | Confounding |
|  | Model 2 with gender | 0.78 (0.39-1.32) | 2 |  | 0.72 (0.39-1.32) | 6 | Not-confounding |
|  | Model 3 with smoking | 0.82 (0.37-1.25) | 3 |  | 0.68 (0.37-1.25) | 2 | Not-confounding |
|  | Model 4 with history of pulmonary diseases | 0.84 (0.39-1.32) | 5 |  | 0.72 (0.39-1.32) | 6 | Not-confounding |
|  | Model 4 with glucocorticoids | 0.84 (0.38-1.29) | 5 |  | 0.70 (0.38-1.29) | 5 | Not-confounding |
|  | Model 5 with benzodiazepine | 0.86 (0.46-1.54) | 8 |  | 0.84 (0.46-1.54) | 20 | Confounding |
|  | Model 6 with neutrophil levels | 0.79 (0.36-1.20) | 1 |  | 0.65 (0.36-1.20) | 3 | Not-confounding |
|  | Model 7 with albumin levels | 0.78 (0.37-1.26) | 2 |  | 0.69 (0.37-1.26) | 2 | Not-confounding |
|  | Model 8 with vitamin D levels | 0.78 (0.36-1.20) | 2 |  | 0.65 (0.36-1.20) | 3 | Not-confounding |
|  | Model 8 with IL-6 levels | 0.74 (0.32-1.10) | 7 |  | 0.59 (0.32-1.10) | 13 | Confounding |

Data are presented as HR (95% CI).

Abbreviations: BMI: body mass index, IMV: invasive mechanical ventilation, HR: hazard ratio, ICU: intensive care unit

HRs were obtained from the Cox regression analysis

**Supplementary Table 2**: Confounding assessment on the associations of body mass index with re-hospitalization and prolonged stay in ICU and hospital among COVID-19 patients

|  |  | Overweight | |  | Obesity | | Results |
| --- | --- | --- | --- | --- | --- | --- | --- |
|  |  | RR (95 CI) | ΔRR (%) |  | RR (95 CI) | ΔRR (%) |  |
| ICU stay≥7 days | |  |  |  |  |  |  |
|  | Crude model | 0.93 (0.54-1.61) | - |  | 0.96 (0.44-2.12) | - |  |
|  | Model 1 with age | 0.96 (0.54-1.69) | 3 |  | 0.99 (0.44-2.23) | 3 | Not-confounding |
|  | Model 2 with gender | 0.95 (0.55-1.65) | 2 |  | 0.86 (0.39-1.92) | 11 | Confounding |
|  | Model 3 with smoking | 0.94 (0.55-1.62) | 1 |  | 0.95 (0.43-2.10) | 1 | Not-confounding |
|  | Model 4 with history of pulmonary diseases | 0.95 (0.55-1.65) | 3 |  | 1.00 (0.45-2.20) | 4 | Not-confounding |
|  | Model 4 with glucocorticoids | 0.91 (0.53-1.57) | 2 |  | 0.97 (0.44-2.13) | 1 | Not-confounding |
|  | Model 5 with benzodiazepine | 0.95 (0.55-1.64) | 2 |  | 1.04 (0.47-2.31) | 8 | Not-confounding |
|  | Model 6 with neutrophil levels | 0.94 (0.55-1.63) | 2 |  | 0.97 (0.44-2.13) | 1 | Not-confounding |
|  | Model 7 with albumin levels | 0.94 (0.54-1.62) | 1 |  | 0.96 (0.43-2.12) | 0 | Not-confounding |
|  | Model 8 with vitamin D levels | 0.98 (0.56-1.71) | 5 |  | 1.02 (0.46-2.25) | 5 | Not-confounding |
|  | Model 8 with IL-6 levels | 0.94 (0.55-1.63) | 1 |  | 0.97 (0.44-2.14) | 1 | Not-confounding |
| Hospital stay≥14 days | |  |  |  |  |  |  |
|  | Crude model | 1.11 (0.66-1.88) | - |  | 2.00 (0.91-4.40) | - |  |
|  | Model 1 with age | 0.92 (0.53-1.60) | 21 |  | 1.62 (0.72-3.65) | 24 | Confounding |
|  | Model 2 with gender | 1.13 (0.67-1.93) | 2 |  | 1.83 (0.82-4.05) | 10 | Confounding |
|  | Model 3 with smoking | 1.11 (0.65-1.88) | 0 |  | 2.06 (0.93-4.55) | 3 | Not-confounding |
|  | Model 4 with history of pulmonary diseases | 1.07 (0.63-1.83) | 4 |  | 1.90 (0.86-4.21) | 5 | Not-confounding |
|  | Model 4 with glucocorticoids | 1.08 (0.63-1.83) | 3 |  | 2.03 (0.92-4.46) | 1 | Not-confounding |
|  | Model 5 with benzodiazepine | 1.09 (0.64-1.86) | 2 |  | 1.75 (0.78-3.92) | 14 | Confounding |
|  | Model 6 with neutrophil levels | 1.11 (0.66-1.88) | 0 |  | 2.00 (0.91-4.40) | 0 | Not-confounding |
|  | Model 7 with albumin levels | 1.07 (0.63-1.82) | 4 |  | 1.88 (0.85-4.18) | 6 | Not-confounding |
|  | Model 8 with vitamin D levels | 1.23 (0.71-2.11) | 9 |  | 2.20 (0.99-4.89) | 9 | Not-confounding |
|  | Model 8 with IL-6 levels | 1.14 (0.67-1.93) | 2 |  | 2.10 (0.95-4.66) | 5 | Not-confounding |
| Re-hospitalization | |  |  |  |  |  |  |
|  | Crude model | 0.78 (0.42-1.47) | - |  | 0.79 (0.31-2.00) | - |  |
|  | Model 1 with age | 0.85 (0.44-1.65) | 8 |  | 0.87 (0.33-2.27) | 9 | Not-confounding |
|  | Model 2 with gender | 0.79 (0.42-1.48) | 1 |  | 0.73 (0.29-1.88) | 8 | Not-confounding |
|  | Model 3 with smoking | 0.79 (0.42-1.47) | 0 |  | 0.78 (0.31-1.99) | 1 | Not-confounding |
|  | Model 4 with history of pulmonary diseases | 0.80 (0.43-1.50) | 2 |  | 0.82 (0.32-2.08) | 3 | Not-confounding |
|  | Model 4 with glucocorticoids | 0.80 (0.43-1.50) | 2 |  | 0.79 (0.31-2.00) | 0 | Not-confounding |
|  | Model 5 with benzodiazepine | 0.80 (0.42-1.49) | 1 |  | 0.87 (0.34-2.23) | 9 | Not-confounding |
|  | Model 6 with neutrophil levels | 0.79 (0.42-1.49) | 1 |  | 0.79 (0.31-2.00) | 0 | Not-confounding |
|  | Model 7 with albumin levels | 0.81 (0.43-1.53) | 3 |  | 0.84 (0.33-2.14) | 5 | Not-confounding |
|  | Model 8 with vitamin D levels | 0.90 (0.46-1.75) | 13 |  | 0.90 (0.35-2.34) | 12 | Confounding |
|  | Model 8 with IL-6 levels | 0.78 (0.42-1.47) | 0 |  | 0.79 (0.31-2.00) | 0 | Not-confounding |

Data are presented as OR (95% CI).

Abbreviations: BMI: body mass index, ICU: intensive care unit, OR: odds ratio

ORs were obtained from the binary logistic regression
